# Effects of the Cyclin-Dependent Kinase 10 (CDK10) on the Tamoxifen Sensitivity of Keloid Samples

**DOI:** 10.3390/molecules17021307

**Published:** 2012-02-01

**Authors:** Ying Liu, Zhibo Xiao, Daping Yang, Lihong Ren, Guofeng Liu, Lin Yang

**Affiliations:** Department of Plastic and Aesthetic, The Second Affiliated Hospital of Harbin Medical University, Harbin 150086, China; Email: desiree5895@163.com (Y.L.); xiaozhibodoctor@yahoo.com.cn (Z.X.); ren_lihong@yahoo.com.cn (L.R.); liugufeng1974@163.com (G.L.); 395798305@qq.com (L.Y.)

**Keywords:** CDK10, keloid, apoptosis, tamoxifen

## Abstract

Cyclin-dependent kinase 10 (CDK10) is a cell cycle regulating protein kinase, which has just been discriminated in recent years. In this paper, mRNA and protein expression of CDK10 were first investigated by a comparative study between 23 human keloid tissue samples and their adjacent normal skin. To further address its potential as a therapeutic target in the treatment of keloid, a plasmid expressing the CDK10 gene was transfected into keloid fibroblast. The effects on tamoxifen-induced apoptosis were then investigated using Western blot assay and flow cytometry. Results showed that there is a generally down-regulated expression of CDK10 in keloid compared to normal skin samples. Transfection with the recombinant CDK10 plasmid significantly decreased the viability of cells and increased the apoptosis rates. Tamoxifen sensitivity in keloid fibroblasts was observed after treatment with the recombinant CDK10 plasmid. The results suggested that CDK10 may play an important role in enhancement of tamoxifen efficiency, and its expression may have a synergistic effect on keloid treatments.

## 1. Introduction

Keloid can extend beyond the boundaries of the original wound and invade the normal surrounding skin. The clinical appearance of keloid is a raised growth, usually accompanied by pruritus and pain. Since the pathogenesis of keloid is still unknown, keloid healing remains impaired [1]. Development of keloid contains atypical fibroblasts and consists of overabundant extracellular matrix components including collagen, fibronectin and certain proteoglycans [2]. Treatment for keloid is problematic, with no single modality producing uniformly satisfactory results [3].

Tamoxifen [1-(*p*-dimethylaminoethoxyphenyl)-1,2-diphenyl-1-butene], a selective estrogen receptor (ER) modulator, has been widely used for the treatment and prevention of recurrence for patients with hormone receptor (ER or progesterone receptor)-positive breast cancers in more than 120 countries throughout the worldwide [4]. Many studies have shown that the mode of action of tamoxifen is connected with apoptosis. It was found that *in vitro* administration of tamoxifen induced a Bcl-2 up-regulation in breast cancer cells [5,6,7]. The same thing happened in human cholangiocarcinoma cell line QBC939, where an up-regulation of Bcl-2 and a down-regulation of Bax has been found after tamoxifen treatment [8]. Tamoxifen is one of the most successful agents used in the management of hormone receptor positive breast cancer. Recently, it has been suggested that tamoxifen might be a novel option for the clinical modulation of wound healing [9,10,11]. Tamoxifen was originally thought to inhibit cell growth by competitive binding to the estrogen receptor, but it has been shown to inhibit the growth of some estrogen-negative breast cancer cell lines [12]. The benign mesenchymal tumors desmoids, which show low expression in estrogen receptors, have been treated successfully with tamoxifen [13]. It has also been indicated that tamoxifen decreases fibroblast function in Dupuytren’s affected palmar fascia [14] and in retroperitoneal fibrosis [9]. Furthermore, tamoxifen has been approved to reduce proliferation of both keloid and normal dermal fibroblasts [15,16]. Payne [17] stated that down-regulating causes of fibrosis with tamoxifen are a possible molecular approach to treat rhinophyma. Evidence [18] suggests that there was a significant inhibition of keloid fibroblasts by tamoxifen, and tamoxifen concentrations greater than 20 µM had a deadly effect on keloid cells, while concentrations between 8 and 12 µM demonstrated significant inhibition of fibroblast cells (*p* < 0.01). The mechanism of tamoxifen-decreased fibrosis is not entirely understood. 

Cyclin-dependent kinases (CDKs), which belong to a large protein family, have 13 members that have been found so far in human cells, including CDK10 [19]. The function of CDKs10 was proven as an important determinant of resistance to endocrine therapies (tamoxifen) for breast cancer [19]. CDK10 silences increases ETS2-driven transcription of c-RAF, resulting in MAPK pathway activation and loss of tumor cell reliance upon estrogen signaling [18], but to the best of our knowledge, there are still no literature reports on the roles of CDK10 in keloid pathogenesis. 

In this study, first a comparative study of CDK10 mRNA and protein expression in 23 human keloid and adjacent normal skin tissue samples by quantitative real-time PCR and Western blot assay was undertaken. Then, whether CDK10 expression was relevant to tamoxifen sensitivity in keloid was investigated by MTT, flow cytometry and Western blot assay. As far as we know, this is the first report demonstrating the effects of CDK10 on keloid tamoxifen sensitivity.

## 2. Results and Discussion

### 2.1. Expression of CDK10 in Keloid and Normal Skin Samples by Quantitative Real-time PCR and Western Blot Assay

**Figure 1:**
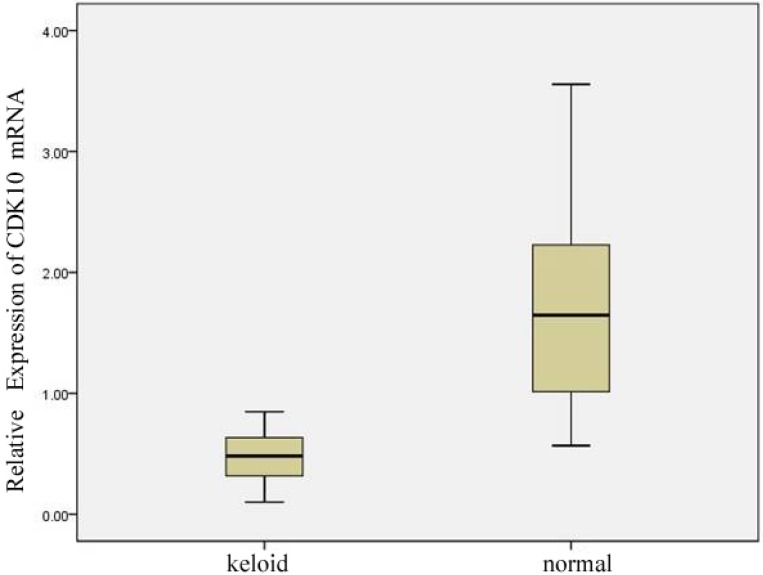
CDK10 expression in keloid and normal skin samples (* means *p*-value of <0.01 compared with normal skin samples).

CDK10 mRNA expression of 23 keloid and normal skin samples was detected by real-time PCR analysis. The mRNA level of CDK10 was noted to be differentially expressed in the keloid and normal skin samples. As shown in Figure 1, CDK10 mRNA levels were significantly higher in the normal skin samples (median 1.72, range 0.57 to 3.56) than in keloid (median 0.47, range 0.10 to 0.85).

**Figure 2:**
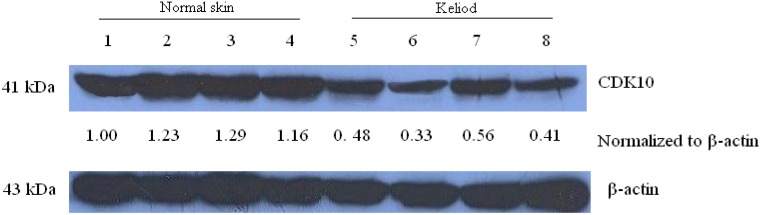
The protein level of CDK10 in keliod and normal skin samples detected by Western blot assay (1–4, normal skin samples; 5–8, keliod samples). The blots were stripped and reprobed with anti-β-actin antibody to normalize the protein loading. Bands were quantitated by densitometric analysis. Fold change represents the protein level of keliod and samples to the first normal skin sample and the resulting protein levels were then normalized to the β-actin protein.

Then CDK10 protein expression of keloid and normal skin samples were checked by Western blot assay (Figure 2). CDK10 protein was greatly decreased in keloid compared with normal skin samples. This result confirmed the lower expression level of CDK10 in keloid samples.

### 2.2. Results of Transfection

**Figure 3:**
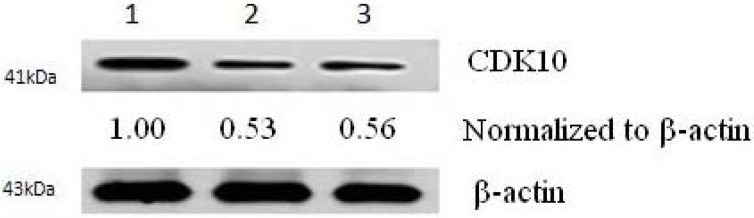
CDK10 protein expression increased notably after pCMV6-CDK10 transfection. Cells were harvested 72 h after transfection; the relative density of bands was quantified by densitometry. The transfected group of CDK10 protein (lane1) demonstrated a visible increase relatively to the empty plasmid transfected (lane 2) or untreated (lane 3) keloid fibroblast cells.

After 72 h transfection of CDK10 with the pCMV6-plasmid and control plasmids, the expression of CDK10 can be detected by Western blot analysis. Western blot analysis for CDK10 revealed that there was a remarkable increase in CDK10 protein expression in CDK10 transfected fibroblasts compared with cells transfected with control plasmid and untransfected controls (Figure 3).

### 2.3. Cytotoxicity Assays

**Figure 4:**
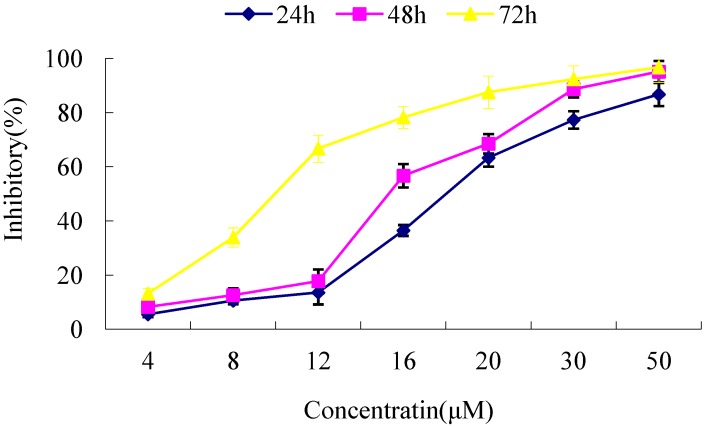
Effect of tamoxifen towards keloid fibroblast as determined by MTT assay.

The MTT method was used to measure the cell optical density of pCMV6-CDK10-transfected fibroblast after treated with various concentrations of tamoxifen (4–50 µM) for 24 h, 48 h and 72 h, respectively. Results showed that tamoxifen inhibited the growth of pCMV6-CDK10-transfected cells in a time- and dose-dependent manner (Figure 4). The IC_50_ values of normal keloid fibroblast cells and transfected keloid fibroblast cells were then compared in the following experiment. The IC_50_ values significantly decreased in the pCMV6-CDK10-transfected cells compared to that of control (*p* < 0.01) (Table 1). All these results showed that CDK10 transfected keloid cells were more sensitive to tamoxifen treatment.

**Table 1:** Inhibition concentrations 50% (IC_50_) values for tamoxifen towards keloid fibroblast cells and pCMV6-CDK10-transfected cells determined by MTT assay. The symbols * indicate significant differences (*p* < 0.01) with respect to control (keloid fibroblast).

Cell lines	IC_50_ (µM)
keloid fibroblast	17.61
pCMV6-CDK10-transfected cells	9.41 *

### 2.4. Keloid Fibroblast Cell Apoptosis as Detected by Annexin V-FITC/PI

**Figure 5:**
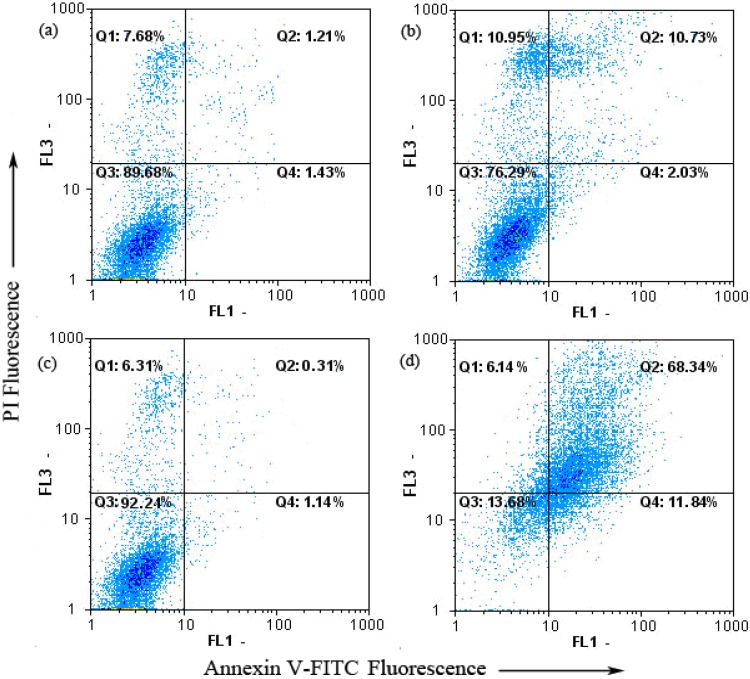
Tamoxifen-induced apoptosis in pCMV6-CDK10-keloid fibroblast using annexinV-FITC/PI. (**a**) Keloid fibroblast treatment with 0 µM tamoxifen; (**b**) Keloid fibroblast treatment with 8 µM tamoxifen; (**c**) pCMV6-CDK10-keloid fibroblast treatment with 0 µM tamoxifen; (**d**) pCMV6-CDK10-keloid fibroblast treatment with 8 µM tamoxifen.

Apoptosis plays an important role in keloid treatment. It is a highly regulated death process by which cells undergo inducible non-necrotic cellular suicide [20]. The Annexin V-FITC apoptosis detection kit was employed to examine the influence of tamoxifen on keloid fibroblast apoptosis by flow cytometry. As shown in Figure 5, only a small percentage of untreated keloid fibroblast (2.64%) cells bound to annexin V-FITC. After treated with tamoxifen, the percentage of annexin V-FITC binding keloid fibroblast cells increased to 12.76%. In contrast, when pCMV6-CDK10-transfected cells treated with tamoxifen, the percentage of annexin V-FITC binding cells increased significantly to 80.18% (*p* < 0.01). To sum up, dots were dispersed and shifted to the Q2 side when pCMV6-CDK10-keloid fibroblast was treated with tamoxifen, indicating that the cells moved to the late apoptotic stage. 

### 2.5. Bax and Bcl-2 Expression of Keloid Fibroblast as Detected by Western Blot

**Figure 6:**
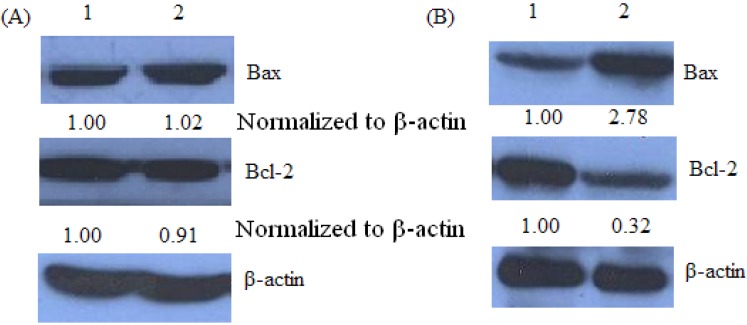
Tamoxifen-mediated up-regulation of Bax and down regulation of Bcl-2 by Western blot assay.Keloid fibroblast cells or pCMV6-C*DK10*-keloid were treated with 8 µMtamoxifen for 48 h, respectively. The blots were stripped and reprobed with anti-β-actin antibody to normalize protein loading. Fold change was calculated as described in Figure 3.

A high Bax/Bcl-2 ratio was clearly correlated with increased apoptotic sensitivity to test reagents [21]. As shown in Figure 6, Western blot analysis revealed a significant increase in the expression of Bax in tamoxifen treated pCMV6-CDK10-keloid fibroblast cells, while there was a significant decrease in Bcl-2 expression, indicating that the Bax/Bcl-2 ratio increased significantly.

## 3. Discussion

Keloid scarring is a disease arising from dermal injury due to a number of aetiologic factors; however, its pathogenesis and optimal clinical treatment remain poorly understood. Many treatments have been used, including surgery, radiation, interferon, laser ablation, steroid injection, and pressure therapy among many others, but few obtain good results and recurrence of keloid remains common [2,3]. 

Cdk10 is a Cdc2-related kinase, previously referred to as PISSLRE, that may play a role in regulating the G2/M phase of the cell cycle [22]. The CDK10 gene was located to chromosome 16q24 in humans. It was first mentioned in 1994 [23] and was noticed to have a close relationship to many cancers. Research showed that the expression of CDK10 was over-expression in lung adenocarcinoma [24] follicular lymphoma [25] and seminomas [26], and low-expression in breast cancer patients who have poorer prognosis after surgical procedure. Iorns *et al*. [27] have shown that CDK10 gene knockdown reproducibly caused resistance to tamoxifen treatment, and these authors also showed that CDK10 could make tamoxifen more effective in treating breast cancer through C-RAF/MAPK kinase pathway activity [27]. Activation of the pathway components ERK1/2 and MEK1/2 was observed following CDK10 silencing, while suppression of ERK pathway signaling at the same time as CDK10 inhibition restored sensitivity to tamoxifen. So far, there is no research showing the expression of CDK10 in keloid tissues. In this study, real-time PCR and Western blot were used to detect the level of CDK10 mRNA and protein level in 23 patients with keloid and adjacent normal skin samples. Results showed us that the relative expressions of CDK10 mRNA and protein level are lower compared with normal skin. Kasten and Giordano [28] noticed CDK10 has been shown to inhibit the transactivation capacity of ETS2, so CDK10 loss would be predicted to potentiate ETS2-mediated gene transcription. By comparing our data with previously published work, we propose that CDK10 determines the response to tamoxifen and other endocrine therapies by modulates intracellular signaling. CDK10 normally binds and represses the ETS2 transcription factor and has an ETS2-binding site in the c-RAF promoter and by using ChIP it was demonstrated that both CDK10 and ETS2 bind to this site [28]. In the absence of CDK10 activity, c-RAF transcription is significantly unregulated due to relief of ETS2 repression. This increase in c-RAF expression leads to activation of downstream components of the MAPK pathway, including MEK1,2 and p42/p44 MAPK, which increase the expression of cyclin D1 [29], resulting in tamoxifen resistance by circumventing the reliance upon estrogen signaling [30]. This evidence may indicate that in keloid the CDK10 may play a role by the same principle.

To investigate the effects of CDK10 on keloid fibroblast, we transfected keloid cells with pCMV-6 CDK10 plasmid to study how CDK10 over-expression affects the sensitivity to tamoxifen treatment, or whether it can make the tamoxifen more effective in keloid cells. Our experimental results demonstrate that pCMV6-CDK10-transfected cells treated with tamoxifen showed a significantly increased apoptosis rate at both the early and late periods (*p* < 0.01) compared with the control group treated with tamoxifen. This result revealed that expression of the CDK10 protein had a synergistic effect on apoptosis in combination with tamoxifen treatment. Data obtained from flow cytometric annexin V-FITC/PI staining showed that tamoxifen induced apoptosis in pCMV6-CDK10-keloid fibroblast. Thus, we further evaluated the expression of the pro-apoptotic protein Bax and the anti-apoptotic protein Bcl-2. These two proteins were crucial determinants of the apoptotic response mediated by many agents. Bcl-2 family proteins play important roles in apoptosis regulation. Anti-apoptotic (Bcl-2 and Bcl-xL, *etc.*) and pro-apoptotic (e.g., Bad, Bax and Bak) are two of the major members in Bcl-2 family [31,32,33]. Anti-apoptotic Bcl-2 and Bcl-xL inhibit apoptosis by sequestering proforms of capsases or by preventing the release of mitochondrial apoptogenic factors [34,35], whereas Bad, Bax and Bak inhibit Bcl-2 activity and promote apoptosis [36]. In this study, tamoxifen treatments altered the expression of anti-apoptotic (Bcl-2) and pro-apoptotic (Bax) proteins, resulting in pCMV6-CDK10-keloid fibroblast cell apoptosis. This result could explain the lower IC_50_ values of tamoxifen-treated pCMV6-CDK10-keloid fibroblast. As such, these data confirmed the synergistic affect of CDK10 expression in combination with tamoxifen treatment on keloid apoptosis rates. Although the mechanism of CDK10 action is unclear, CDK10 can be taken as a potential marker for sensitivity in prospective clinical trials of keloid patients treated with tamoxifen therapies. Further studies are demanded to further discuss the mechanism of CDK10 and the relationship with tamoxifen in keloid cells.

## 4. Experimental Section

### 4.1. Patients and Treatment

Keloid and normal skin tissue samples were obtained from 23 patients who underwent surgeries from 2009 to 2011 at the Second Affiliated Hospital of Harbin Medical University. Informed consent was obtained from each patient recruited, and the study was approved by the Hospital Ethics Committee. Keloid cells were obtained at surgical release from six patients aged 18–37 years who had a non-peduncle keloid on the manitrunk, ear lobe and upper arm of at least 1-year evolution, with clinical activity such as growth, hyperaemia, pruritus and pain. Primary cultures of fibroblasts from the surgical specimens were then established. Cells from passages 3 to 8 were used for experiments. Cell was maintained in Dulbecco’s modified Eagle’s medium (DMEM; Gibco BRL, Grand Island, NY, USA). All cell lines were supplemented with 10% fetal bovine serum (FBS) and 5 mmol/L L-glutamine in a 5% CO_2_ air incubator at 37 °C. Cells were transient transfected with pCMV6-CDK10 or a control plasmid using GeneJuice® Transfection Reagent (Novagen) according to the manufacturer’s protocol.

### 4.2. RNA Extraction and Quantitative Real-time PCR

Total RNA was extracted from the tissue samples with the RNApure Tissue Kit (CWBIO, CW0584). Quantitative real-time PCR (qPCR) was performed using the PrimeScript™ RT reagent Kit (Takara, DRR037A). The qPCR reactions were carried out by SYBR green PCR master mix (Takara, DRR083M) with an Multiplex Quantitative PCR System (Applied Bios stem), and β-actin was used as an internal standard. Primers were designed for qPCR from Primer Express software (Applied Bios stems). The primer sequences employed were: CDK10, forward: 5'-TGGACAAGGAGAAGGATG-3', reverse: 5'-CTGCTCACAGTAACCCATC-3'; β-actin, forward: 5'-AGAAGGAGATCACTGCCCTGGCACC-3' reverse: 5'-CCTGCTTGCTGATCCACATCTGCTG-3'. The PCR cycling conditions were as follows: 10 min at 95 °C, 40 cycles of 30 s at 95 °C, 30 s at 54 °C, and 30 s at 72 °C; and finally 5 min at 72 °C. Melting curve analysis was conducted to determine the specificity of the reaction. Probe experiments showed that the efficiencies of amplification of the primers for the target and reference genes were approximately equal. Each sample was tested in triplicate. A DNA dissociation curve was produced to confirm the specificity of the amplification after the thermal cycling. Relative expression level changes were calculated according to 2-⊿Ct(⊿Ct = Ct[CDK10] − Ct[β-actin]) method as described previously [37]. 

### 4.3. Western Blot Analysis

Total protein extracts of the keloid and normal skin samples and cells were prepared by homogenization in RIPA (Beyotime, P0013B). Briefly, for isolation of total protein fractions, cells or tissue samples were collected, washed twice with ice-cold PBS, and lysed using cell lysis buffer [20 mM Tris pH 7.5, 150 mM NaCl, 1% Triton X-100, 2.5 mM sodium pyrophosphate, 1 mM EDTA, 1% Na_2_CO_3_, 0.5 µg/mL leupeptin, 1 mM phenylmethanesulfonyl fluoride (PMSF)]. The lysates were collected by scraping from the plates and then centrifuged at 12,000 rpm at 4 °C for 15 min. Total protein samples (20 µg) were loaded on a 12% of SDS polyacrylamide gel for electrophoresis, and transferred onto PVDF transfer membranes (Millipore, Billerica, MA, USA) at 0.8 mA/cm^2^ for 70 min. Membranes were blocked at room temperature for 2 h with blocking solution (1% BSA in PBS plus 0.05% Tween-20). Membranes were incubated overnight at 4 °C with primary antibodies (anti-â-actin and anti-Bax were mouse polyclonal antibodies; anti-Bcl-2 and anti-CDK10 were rabbit polyclonal antibodies) at a 1:1,000 dilution (Biosynthesis Biotechnology Company, Beijing, China) in blocking solution. After thrice washings in TBST for each 5 min, membranes were incubated for 1 h at room temperature with an alkaline phosphatase peroxidase-conjugated anti-mouse secondary antibody at a dilution of 1:500 in blocking solution. Detection was performed by the BCIP/NBT Alkaline Phosphatase Color Development Kit (Beyotime Institute of Biotechnology) according to the manufacturer’s instructions. Bands were recorded with a digital camera (Nikon, Tokyo, Japan). 

### 4.4. Transient Transfection

The pCMV6-CDK10 expression plasmid was acquired from Doctor Zhong Xiangyu (Department of General Surgery, the Second Affiliated Hospital of Harbin Medical University, China). The plasmids were sequenced from OriGene Company (Rockville, MD, USA). Plasmid DNA from *Escherichia coli* cell lysates was extracted and purified using a PureLinkTM Hipure Plasmid DNA Purification Kit (Invitrogen, Carlsbad, CA, USA). Keloid fibroblast was transfected with pCMV6-CDK10 using GeneJuice® Transfection Reagent (Novagen) according to the manufacturer’s protocol. In brief, cells were trypsinized and plated onto six-well plates. Then, transfection reagent was added and incubated at room temperature for 5 min. The appropriate volume of plasmid DNA was then added and the cells were incubated for an additional 15 min. The common complete medium was replaced by the antibiotics and serum-free medium. Six hours after the transfection, the medium was replaced by the common complete medium again. After 24, 48 and 72 h the transfection, the cells were then prepared for Western blot analysis, MTT assays, or flow cytometry. 

### 4.5. Cytotoxicity Assay

Inhibition of cell proliferation of tamoxifen (SIGMA) was measured by MTT assay [38]. Briefly, keloid fibroblast was plated in 96-well culture plates (1 × 10^5^ cells/well) separately. After 24 h incubation, normal keloid fibroblast cells was treated with tamoxifen (4, 8, 12, 16, 20, 30 and 50 µM, eight wells per concentration) for 72 h. pCMV6-CDK10-keloid fibroblast cells was treated with tamoxifen (4, 8, 12, 16, 20, 30 and 50 µM, eight wells per concentration) for 24, 48 h or 72 h. MTT solution (5 mg/mL) was then added to each well. After 4 h incubation, the formazan precipitate was dissolved in dimethyl sulfoxide (100 µL), and then the absorbance was measured in an ELISA reader (Thermo Molecular Devices Co., Union City, NJ, USA) at 570 nm. The cell viability ratio was calculated by the following formula: Inhibitory ratio (%) = [(OD_control_ − OD_treated_)/(OD_control_)] × 100%. 

### 4.6. Flow Cytometric Analysis of Cell Apoptosis

The extent of apoptosis was measured through annexinV-FITC apoptosis detection kit (Beyotime Institute of Biotechnology, Shanghai, China) as described by the manufacturer’s instructions. After exposure to tamoxifen for 24 h, cells were collected, washed twice with PBS, gently resuspended in annexin V binding buffer and incubated with annexin V-FITC/PI in dark for 15 min and analyzed by flow cytometry using FloMax software. The fraction of cell population in different quadrants was analyzed using quadrant statistics. The lower left quadrant contained intact cells; lower right quadrant apoptotic and in the upper right quadrant necrotic or post-apoptotic cells. 

### 4.7. Statistical Analysis

The data were expressed as mean ± S.D. All statistics were calculated using the STATISTICA program (Stat Soft, Tulsa, OK, USA). A *p*-value of <0.01 was considered as significant.

## 4. Conclusions

In the present study, a lower expression of CDK10 was found by a comparative study of mRNA and protein expression in 23 human keloid and adjacent normal skin tissue samples by quantitative real-time PCR and Western blot assay. To further determine the potential of CDK10 as a therapeutic target for tamoxifen, CDK10 was over expressed in keloid fibroblast cells and the presence of apoptosis was detected by flow cytometry and Western blot assay.

The apoptosis rate of the combination treatment (tamoxifen combined with pCMV6-CDK10) increased when compared with pCMV6-CDK10 or tamoxifen alone. Further, the present study showed that the increased expression of CDK10 significantly enhanced the effects of tamoxifen treatments on Bax and Bcl2. The findings indicated that CDK10 is an attractive therapeutic target because of its ability to suppress keloid fibroblast growth and enhance tamoxifen sensitivity in keloid.
